# Beyond Vaccines: Clinical Status of Prospective COVID-19 Therapeutics

**DOI:** 10.3389/fimmu.2021.752227

**Published:** 2021-10-01

**Authors:** Sriram Kumar, Duygu Merve Çalışkan, Josua Janowski, Aileen Faist, Beate Claudine Gisela Conrad, Julius Lange, Stephan Ludwig, Linda Brunotte

**Affiliations:** ^1^ Institute of Virology, University of Münster, Münster, Germany; ^2^ EvoPAD Research Training Group 2220, University of Münster, Münster, Germany; ^3^ SP BioSciences Graduate Program, University of Münster, Münster, Germany; ^4^ CiM-IMPRS Graduate Program, University of Münster, Münster, Germany; ^5^ Interdisciplinary Centre for Medical Research, University of Münster, Münster, Germany

**Keywords:** SARS-CoV-2, COVID-19, interferons, antivirals, immunomodulators, Long COVID

## Abstract

Since November 2019 the SARS-CoV-2 pandemic has caused nearly 200 million infection and more than 4 million deaths globally (Updated information from the World Health Organization, as on 2^nd^ Aug 2021). Within only one year into the pandemic, several vaccines were designed and reached approval for the immunization of the world population. The remarkable protective effects of the manufactured vaccines are demonstrated in countries with high vaccination rates, such as Israel and UK. However, limited production capacities, poor distribution infrastructures and political hesitations still hamper the availability of vaccines in many countries. In addition, due to the emergency of SARS-CoV-2 variants with immune escape properties towards the vaccines the global numbers of new infections as well as patients developing severe COVID-19, remains high. New studies reported that about 8% of infected individuals develop long term symptoms with strong personal restrictions on private as well as professional level, which contributes to the long socioeconomic problems caused by this pandemic. Until today, emergency use-approved treatment options for COVID-19 are limited to the antiviral Remdesivir, a nucleoside analogue targeting the viral polymerase, the glucocorticosteroide Dexamethasone as well as neutralizing antibodies. The therapeutic benefits of these treatments are under ongoing debate and clinical studies assessing the efficiency of these treatments are still underway. To identify new therapeutic treatments for COVID-19, now and by the post-pandemic era, diverse experimental approaches are under scientific evaluation in companies and scientific research teams all over the world. To accelerate clinical translation of promising candidates, repurposing approaches of known approved drugs are specifically fostered but also novel technologies are being developed and are under investigation. This review summarizes the recent developments from the lab bench as well as the clinical status of emerging therapeutic candidates and discusses possible therapeutic entry points for the treatment strategies with regard to the biology of SARS-CoV-2 and the clinical course of COVID-19.

## SARS-CoV2 Replication Cycle

Severe Acute Respiratory Syndrome Coronavirus 2 (SARS-CoV-2) is a positive-sense, single-stranded RNA virus that belongs to the lineage *β-coronavirus-2b* the family *Coronaviridae* and the order *Nidovirales* (1). The name coronavirus is derived from the crown-like appearance of the spike proteins of the virus particles under an electron microscope. The viral genome measures approximately 30 kb, and contains fourteen Open Reading Frames (ORF) coding for twenty-seven viral proteins, some of which are not-yet functionally characterized. The virion consists of four structural proteins: Spike (S), Envelope (E), Membrane (M), and Nucleocapsid (N). The S protein, located on the virion surface, primarily binds to the cell-surface receptor Angiotensin Converting Enzyme 2 (ACE2). Due to its strong antigenicity and indispensable role during virus entry, the S protein serves as a target for antiviral treatments and vaccine design. It contains two functional subunits: The S1 subunit binds to and interacts with the cell-surface receptors, while the S2 subunit mediates membrane fusion (2, 3). Alongside S protein binding, cleavage of S proteins at the polybasic cleavage-sites by the membrane-associated Transmembrane Protease Serine 2 (TMPRSS2) and the lysosomal cysteine protease Cathepsin-L facilitates membrane-fusion and virus entry at the cellular endosomes (4, 5). The released viral genomic RNA (gRNA), which carries a cap-structure and a polyA-tail, serves as a direct template for the cellular ribosome-mediated translation of the two large ORFs: ORF1a and ORF1b. The polyprotein products pp1a and pp1ab are independently cleaved by the viral proteases nsp3 and nsp5, which are also prominent drug targets, and post-translationally processed into sixteen non-structural proteins (nsps). Of these sixteen nsps, nsp12 assembles the other nsps to constitute the viral replication-transcription machinery, and also confers catalytic activity to the RNA-dependent RNA polymerase (RdRp), which then catalyzes genome replication and RNA processing (6). The encapsidated progeny gRNAs are then assembled into viral particles in the Endoplasmic Reticulum (ER)-Golgi complex, together mediated by the membrane proteins. The fully assembled virus particles then bud out of the ER-Golgi complex and are released out of the host-cell by membrane exocytosis.

## COVID-19 Clinical Manifestation

SARS-CoV-2 is mainly transmitted by aerosols and infects the cells in the upper respiratory tract (7). However, depending on the viral load, replication speed and local immune response, it is further disseminated to the lower respiratory tract and other organs within days or weeks post infection, by which it is reported to cause severe damage to lungs, stomach, intestine, kidney, heart, blood vessels, liver, brain and skin (8–10). Early symptoms appear after an incubation period of 1-14 days but many infected individuals can remain asymptomatic despite high viral loads, which promotes viral transmission even before the clinical diagnosis of positive infection. Most common early symptoms include fever, dry cough, myalgia, fatigue, and loss of smell and taste. In addition, some patients manifest sputum production, hemoptysis, headache, sore throat, chest pain and gastrointestinal symptoms like diarrhea, nausea and vomiting (7, 11, 12). Dyspnea and pneumonia can develop in a median time span of eight days after symptom onset. In laboratory examinations, leukopenia, lymphopenia, anemia, thrombocytopenia, elevated ferritin and d-dimer level such as an elevated CRP, which were recognized to correlate with severity. Likewise, elevated plasma concentrations of pro-inflammatory cytokines IL-2, IL-6, IL-7, IL-10, GCSF, IP10, MCP1, MIP1A, and TNF-α are predictive for a severe outcome (11, 13). Due to the lack of wide-spread pre-existing adaptive immunity towards the newly emerged zoonotic SARS-CoV-2 virus, the human innate immune response poses the first critical barrier against the infection and is an important determinant of the disease trajectories.

In general, the immune response to SARS-CoV-2 infection can be broadly described in three phases (14). The early phase is predominated by virus replication in the upper respiratory epithelium, but also involve the intestinal epithelium and vascular endothelium, due to the high expression of ACE2 receptors in these linings (15, 16). This phase is often accompanied by mild respiratory and systemic symptoms. The quality and potency of the induced early immune response is critical in this phase and decisive for further disease development. The second phase is characterized by both, viral replication and development of lung inflammation with massive recruitment of cytokine-expressing immune cells to the site of infection. Patients often experience first signs of hypoxia and are admitted to hospital during this phase. The third phase describes severe COVID-19 and is specified by systemic hyper-inflammation with high blood levels of inflammatory cytokines. The systemic involvement causes symptoms like septic shock, respiratory failure, cardiopulmonary collapse and may even lead to multi-organ dysfunction and death (11).

Even though all age groups can be infected by SARS-CoV-2, particularly elderly patients have an increased risk of mortality. Comorbidities such as obesity, hypertension, chronic obstructive pulmonary diseases (COPD), diabetes, cardiovascular and cerebrovascular diseases have been reported as risk factors for severe COVID-19 (10, 17). These patients required more intensive care and experienced more often complications like Acute Respiratory Distress Syndrome (ARDS), coagulation dysfunction, sepsis, and death (10–12, 18). Additionally, immunosuppressed patients as well as cancer patients have been reported as a highly vulnerable group due to low protective antibody responses (19, 20).

Clearly, these dynamics in viral replication and immune activation as well as the patient’s individual constitution require specified therapeutic strategies that are aligned to the disease phase and time of treatment onset. Here, we summarize the current state of development and experiences from first in-human applications of the many antiviral and immunomodulatory drugs that are under investigation in single or combinatorial approaches for the treatment and prevention of COVID-19.

## Direct-Acting Antivirals (DAA)

According to their essential enzymatic functions for the replication of SARS-CoV-2, the S protein, the viral polymerase as well as the viral proteases are the most prominent targets for diverse virus-targeting antiviral strategies.

### Umifenovir

Umifenovir (Arbidol) has been previously applied against some enveloped and non-enveloped viruses especially Influenza A and B, and recently against hepatitis C virus (HCV) (21). Although the mechanism of action of Umifenovir is not exactly understood, it was suggested to inhibit trimerization of the SARS-CoV-2 S protein (22). Molecular simulations indicated that it stabilizes the RBD-ACE2 complex, thus limiting conformational rearrangements associated with membrane fusion and virus entry (23). While some clinical studies showed that Umifenovir positively affects patient recovery and mortality, other studies reported that treatment of patients not requiring intensive care does not show different results than the control group in terms of recovery and virus clearance (24–26). A study comparing Arbidol and Lopinavir/ritonavir (LPV/r) treatments reported that Arbidol had a significantly positive effect on clinical and laboratory improvements on COVID-19 patients (24, 25). Another study comparing Arbidol and LPV/r combined therapy with LPV/r therapy alone shows that the group receiving the combination therapy was associated with a higher rate of coronavirus test negative conversions rate on day 7 (combination group %75, control group %35) and on day 14 (combination group %94, control group %52,9) and significant improvement in chest CT scans (combination group %69, control group %29) at day 7 (24, 25). In contrast, a meta-analysis of 137 reports on the efficacy and safety concludes that Umifenovir is safe, led to higher negative PCR rates after 14 days but had no positive effects on patient recovery (27). Despite these contradictory results, further combinatorial studies with different antivirals and interferons (IFN) are planned (Clinical study identifier: NCT04350684, NCT04273763).

### Clofazimine

Clofazimine is an orally available and FDA (Food and Drug Administration)-approved anti-leprosy drug and has been shown to reduce SARS-CoV-2 infection by inhibiting spike-dependent cell fusion and the activity of the viral helicase in many *in vitro*, *ex vivo* and *in vivo* systems (28). Interestingly, it demonstrated synergistic antiviral activity in combination with Remdesivir. A Phase-II clinical trial (NCT04465695) is ongoing, evaluating dual therapy with Clofazimine and IFN-β1b for treatment of hospitalized COVID-19 patients in China.

### Convalescent Sera and Monoclonal Antibodies

Like for other virus infections, the therapeutic value of convalescent sera that contain neutralizing antibodies to SARS-CoV-2 was evaluated. Although early clinical studies demonstrated improvement of symptoms and viral clearance within an average of 5-6 days in patients receiving convalescent plasma treatment (29–32), results from recent large-scale trials, such as the RECOVERY Collaborative Group, report the absence of positive outcomes (33, 34). With respect to the general associated risks of convalescent plasma therapy regarding blood product transfusion (e.g. blood-borne infections, allergic reactions), transfusion-related acute lung injury and the potential for antibody-dependent infection enhancement (ADE), this treatment option is considered with care (35). Importantly, convalescent sera treatment of immunosuppressed patients with chronic SARS-CoV-2 infections resulted in the evolution of virus variants with immune escape mutations (36). Similar problems occurred during treatment of patients with mild to moderate COVID-19 with the monoclonal antibody Bamlavinimab that targets the S protein Receptor Binding Domain (RBD) and was given Emergency Use Authorization (EUA) in November 2020. However, due to complications and rapid resistance development, the EUA was withdrawn in April 2021 (FDA News release, April 2021). More promising is the use of monoclonal antibody cocktails. Combination of Bamlanivimab and Etesevimab, which are both human immunoglobulin G1 (IgG1) kappa antibodies that target different epitopes of the SARS-CoV-2 spike protein (37, 38) showed a significant decrease in viral load in nose and upper respiratory tract on the 11th day post treatment in a randomized Phase-II study performed with 577 mild/moderate COVID-19 patients (39). The combination was given EUA by the FDA for the use in patients with a high risk of developing severe COVID-19. Also, the European Medicines Agency (EMA) initiated a rolling review process for approval.

Another antibody cocktail named REGN-COV2 contains the two non-competing neutralizing antibodies Casirivimab and Imdevimab, both targeting the S protein RBD (40). Results from a clinical study with 275 patients showed that REGN-COV2 reduced viral loads with a greater effect in patients whose immune response was not yet initiated or had a high initial viral load. No side effects were seen and its use was considered safe (41). In addition, recent clinical trials tested use of REGN-COV2 for preventive treatment. This demonstrated 73% protection of household contacts from symptomatic infections (NCT04452318). Another approach is based on an engineered, double acting recombinant human antibody VIR-7831 (Sotrovimab). This engineered antibody is derived from a parental antibody (S309) that was isolated from the memory B cells of a SARS-CoV-2 survivor. Sotrovimab targets a highly conserved glycan in the S protein that is part of a region not competing with ACE2 binding and does not cover any of the reported positions that are mutated in the current SARS-CoV-2 variants of concern. To improve serum half-life and mucosal distribution, Sotrovimab contains several modifications in the Fc region. In addition, VIR-7832, which is otherwise identical to VIR-7831, contains a GAALIE mutation to promote the induction of CD8+ T-cells during viral respiratory infections. Importantly, both antibodies demonstrated efficient neutralization of SARS-CoV-2 including the Alpha, Beta and Gamma variants in the context of a VSV-pseudotype model (42). EUA application was filed to the FDA for VIR-7831 in March 2021 according to the observed 85% reduction of hospitalization or death in Phase-III clinical trials and EMA has started a rolling review for treatment of patients with mild to moderate COVID-19, which do not require oxygenation but are at risk to develop severe disease. In addition, VIR-7831 is currently investigated in a randomized multi-centre Phase-II/III clinical study for treatment of mild COVID-19 in outpatients (NTC04545060).

### Human Recombinant Soluble ACE2 (hrsACE2)

ACE2 is the major receptor for SARS-CoV-2 and a regulator of the renin-angiotensin system that protects many tissues, including the lung, from injuries and has a role on blood pressure regulation and electrolyte homeostasis. Because of these functions, it is seen as a good therapeutic candidate for COVID-19 by using it as a decoy receptor and immune regulator (43, 44). A soluble version of the human ACE2 protein (hrsACE2, APN01) reduces the SARS-CoV-2 viral load 1000-5000- fold in cell cultures and shows an inhibitory effect on cell attachment in human blood vessel and kidney organoids. In addition, the combination therapy with Remdesivir and human soluble ACE2 reduced the dose of Remdesivir and human ACE2 required for treatment, as it inhibited both, the binding of the virus to the cell and its replication (45). Clinical studies confirmed safety and tolerability of hrsACE2 and demonstrated clinical benefits, such as more ventilation-free days and reduced RNA loads (NCT00886353, NCT01597635) (NCT04335136) (45). Based on these positive outcomes it was announced that APN01 will be included in the large-scale NIH-funded ACTIV Phase-II clinical trial (ACTIV-4d RAAS). Furthermore, combination of recombinant soluble human ACE with Remdesivir was shown to enhance its antiviral effects *in vitro* (46). An alternative approach utilizes the therapeutic molecule obtained by binding the human Fc region to the ACE2 receptor by antibody engineering techniques (47). Fusion of the human IgG Fc moiety to the ACE2 ectodomain moiety results in an extended half-life. A version of ACE2 IgG1 Fc is currently evaluated in a Phase-I clinical trial. Developing this therapeutic candidate, it has been optimized with IgG4-Fc to facilitate Fc receptor activation and prevention of antibody-dependent disease development (48).

### Remdesivir

Remdesivir is metabolized in host cells to form a nucleoside triphosphate that competes with ATP for incorporation into progeny viral RNA by the viral polymerase (4) and has proven effectiveness against coronaviruses *in vitro* and *in vivo* (49–51). Studies of the ACTT-1 trial (NCT04280705) showed that Remdesivir shortens the recovery time (median 11 days compared to 15 days in the placebo group) (52, 53) of ventilated COVID-19 patients and was granted EUA for use in severe COVID-19 patients in the US and the EU. Considering the results of other clinical studies, FDA gave full approval of Remdesivir for COVID-19 treatment a few month later in the US (52–54). However, the WHO SOLIDARITY study reported that Remdesivir had little or no effect on the length of hospitalization, transition to ventilation, or overall mortality and recommended against the use of it for COVID-19 treatment (55). In line with this study, in patients with mild to moderate COVID-19 without respiratory support, Remdesivir does not provide a significant benefit but can shorten the recovery time in patients with an early diagnosis (≤10 days) (56). Despite the controversial results of the available clinical trials, Remdesivir is widely used as standard care in combination with dexamethasone for critically ill COVID-19 patients (56). Further clinical studies evaluating time of application, effect on comorbidities and diverse drug combination are still ongoing.

### Favipiravir

Favipiravir is a prodrug and acts by transforming into a metabolite of ribofuranosyl 5’triphosphate (57) that inhibits viral RNA polymerases (58, 59). It is effective against many RNA viruses such as Lassa, Marburg and Nipah virus, and is approved for the treatment of influenza in Japan (60). Many clinical studies have assessed the effectiveness of Favipiravir for treatment of COVID-19 and it was approved in several countries. However, data availability of the results of these studies is difficult preventing proper meta-analysis (61). Previous studies reported teratogenicity and embryotoxicity in different study models, thus use in pregnant women should be avoided (60, 62). Clinical studies in patients with mild and moderate COVID-19, comparing Favipiravir, Umifenovir and LPV/r treatments have shown that time of viral clearance and recovery of radiological findings are faster in patients using Favipiravir (63, 64). In a clinical study investigating the effectiveness of chloroquine and Favipiravir, no significant difference was found between the two drugs (65). Currently, clinical studies for at-home medication are underway within the PRINCIPLE trial (66).

### Molnupiravir

Molnupiravir (MK-4482) is an orally available nucleoside analogue with similar mechanism to Favipiravir and inhibits effectively SARS-CoV-2 in *in vitro* and *in vivo* (67, 68). It is currently evaluated in Phase-II/III trials for outpatients and inpatients with COVID-19 (NCT04405739, NCT04405570, NCT04575584). In combination with Favipiravir in hamster models of SARS-Cov-2, Molnupiravir was able to reduce the viral load in the lungs of infected hamsters by approximately 5 log10 (68, 69). Molnupiravir increased the frequency of mutations in MERS-CoV viral RNA in infected mice, and in the SARS-Cov-2 treatment study, the number of these mutations in the Molnupiravir/Favipiravir combination group was found to be significantly higher than that in the highest dose group of Molnupiravir alone. These results indicate a similar mode of action for the marked decrease in infectious viral loads observed in the combination therapy group for SARS-CoV-2 (68).

### Lopinavir/ritonavir (LPV/r)

LPV/r is a broad-spectrum protease inhibitor used in the treatment of AIDS. Due to its low bioavailability when taken orally, it is used in combination with the pharmacokinetic enhancer ritonavir. LPV/r is supposed to target the SARS-CoV-2 C-like proteinase (3CLpro) substrate binding site, which is highly conserved among all coronaviruses (69). Despite this expected broad-spectrum effect, clinical studies have not shown a significant difference in clinical improvement between patients receiving standard therapy with/without LPV/r or Umifenovir (70, 71). Although the results have reduced the trust in LPV/r therapy, other studies reported that adding ribavirin and IFN-β1b to LPV/r therapy results in faster recovery and virus clearance in SARS-CoV-2 patients (72, 73). Most patients using LPV/r experienced non-serious side effects (mostly gastrointestinal problems). However, it has been reported in clinical studies that some patients were unable to continue treatment due to serious side effects (74). In addition, it is known that it may cause hypertriglyceridemia, hypercholesterolemia, pancreatitis, hepatotoxicity and cardiac problems. The use of LPV/r, especially with drugs that prolong the QT interval (time from the start of a Q wave to the end of the next T wave in an electrocardiogram), which describes the time taken by the cardiac ventricles to depolarize and repolarize, is one of the important issues to be considered (75).

## Host-Directed Antivirals (HDA)

All viruses, including SARS-CoV-2, exploit the host cell resources to replicate and ensure transmission to other hosts. Virus-host interactions occur at all stages of the viral life cycle and provide numerous factors that are crucial for virus survival and therefore may represent targets for antiviral approaches. A major advantage of targeting the host rather than the virus is the low risk of resistance development and the high potential of broad use against other pathogens that rely on related cellular proteins or processes.

### (Hydroxy-) Chloroquine

Huge efforts have been taken to systematically screen existing drug libraries in order to find treatments for COVID-19 (76). This revealed a number of repurposed candidates such as (Hydroxy-) Chloroquine (HCQ), which has previously shown beneficial effects in the treatment of malaria and autoimmune diseases and displayed controversially discussed antiviral properties against HIV and SARS-CoV (77). HCQ increases the pH of cellular endosomes and vacuoles and thereby affects the activity of many enzymes, including Cathepsin L, which is needed to mediate cleavage of the SARS-CoV-2 S protein, followed by membrane fusion and release of the viral genome into the host cell (78, 79). Even though chloroquine exhibited antiviral effects against both SARS-CoV and SARS-CoV-2 *in vitro*, and also in mice using the common-cold corona virus OC43 (80–83), all clinical trials failed to protect patients from severe COVID-19 (84–86). This discrepancy might be explained by the dependency of SARS-CoV-2 on another protease, namely TMPRSS2, which is important for virus entry and remains unaffected by HCQ (79). Due to the lack of efficacy, the FDA revoked the EUA for HCQ in June 2020 (87).

### Camostat and Nafamostat

Direct targeting of the host cell protease TMPRSS2, which cleaves and thus activates the S protein (88, 89), Camostat mesylate or its active metabolite 4-(4-guanidinobenzyloxy)phenylacetic acid (GBPA) demonstrated efficient antiviral activity against SARS-CoV and SARS-CoV-2 *in vitro* (4, 90). Currently, Camostat, which is approved in Japan for the treatment of chronic pancreatitis, is evaluated in diverse international clinical trials of all phases for the treatment of hospitalized and non-hospitalized COVID-19 patients in various disease stages and comorbidities for single or combination treatments with other drugs (NCT04455815, NCT04321096) (91).

In a comparative *in vitro* study with Nafamostat mesylate, another protease inhibitor targeting TMPRSS2, inhibition of SARS-CoV-2 entry was 15-fold higher compared to treatment with Camostat mesylate. In addition, Nafamostat mesylate showed higher inhibition of SARS-CoV-2 infection *in vitro* (92). Several Phase-II/III clinical trials with Nafamostat mesylate for treatment of hospitalized COVID-19 patients are currently ongoing (NCT04352400, NCT04473053, NCT04623021). However, whether the required concentrations of these inhibitors can be attained in the human lung remain to be determined (93). Nevertheless, these studies demonstrate that targeting the main host proteases involved in the virus lifecycle provides a promising approach to inhibit viral replication and thus have initiated numerous studies that aim to characterize host protease in various stages of virus infection, as reviewed previously (94, 95).

### Fluoxetine

Recently, the therapeutic effect of Functional Inhibitors of Acid Sphingomyelinase (FIASMA) such as the antidepressant Fluoxetine to inhibit SARS-CoV-2 cell entry has been investigated by several groups. This revealed, that Fluoxetine treatment efficiently reduced viral entry and replication without cytotoxic effects *in vitro* (96, 97). A follow-up study further showed pronounced drug synergism of Fluoxetine and Remdesivir, thereby providing additional therapeutic options for COVID-19 treatment (98). In addition to endolysosomal acidification, Fluoxetine was shown to disrupt the NFkB/IL-6 axis, which is associated with cytokine-induced pathologies during COVID-19 (99). On this basis, a pharmacokinetic study estimated efficient inhibition of SARS-CoV-2 at a commonly well-tolerated concentration of Fluoxetine used for treatment of depression (100). A retrospective observational study investigated a potential beneficial effect of anti-depressant intake, including Fluoxetine, during COVID-19. The results indicated a significant reduction in the risk of intubation and death in hospitalized COVID-19 patients (101). Furthermore, an ongoing Phase-IV clinical trial investigates the potential of Fluoxetine treatment to reduce intubation and death from SARS-CoV-2 infection (NCT04377308).

### Plitidepsin

For translation of viral proteins, SARS-CoV-2 relies on the host cellular translation machinery. Recently, a well-described inhibitor of the eukaryotic translation elongation factor 1 alpha (eEF1A), called Plitidepsin (Brandname: Aplidin) was discovered and characterized to have potent antiviral effects against SARS-CoV-2 (102–104). Intriguingly, Plitidepsin inhibited virus infections more efficient than Remdesivir by a factor of 27.5 *in vitro*. Moreover, SARS-CoV-2 titers in lungs of mice expressing human ACE2 were significantly reduced upon prophylactic treatment with Plitidepsin, facilitated by eEF1A inhibition (105). Furthermore, it exhibits antiviral potential against the SARS-CoV-2 Alpha variant (106). Plitidepsin has originally been developed for the treatment of multiple myeloma (103). Interestingly, in earlier studies with myeloma patients, Plitidepsin has also been used in combination with the anti-inflammatory drug dexamethasone (107). This provides already existing data for drug tolerance in combination therapies of dexamethasone and Plitidepsin in COVID-19 patients as well. However, EMA refused marketing authorization of Plitidepsin as an anti-cancer medication twice, in 2017 and 2018. The safety and tolerability of Plitidepsin was recently evaluated in COVID-19 patients in a Phase-I trial (NCT04382066) but results have not been released yet. In addition, a Phase-III trial for use of Plitidepsin in patients with moderate COVID-19 was filed but not yet started (NCT04784559).

### MEK Inhibitor ATR-002

Host kinases represent another group of cellular targets against SARS-CoV-2 infection (108). The RAF/MEK/ERK-signaling cascade is known to be a central pathway involved in virus replication of several RNA viruses, including influenza, RSV and coronavirus (109–112). Previous studies using the MEK inhibitor ATR-002 demonstrated reduced influenza viral titers by preventing export of viral ribonucleoprotein complexes during the viral infection cycle (109, 113). Moreover, there are indications that treatment with ATR-002 reduced cytokine expression, such as TNF-α, IL-1β, and IP-10 *in vitro* and *in vivo* and thereby could help to counteract or rebalance the SARS-CoV-2 induced hyperactivation of the immune response (114). A Phase-I clinical trial demonstrated that ATR-002 is safe and very well tolerated in humans with little or no drug related side effects (NCT04385420). Currently, ATR-002 is tested in a Phase-II clinical trial RESPIRE for treatment of COVID-19 (NCT04776044).

### Ivermectin

Ivermectin is an antiparasitic drug that has been used for years, thus providing well described pharmacokinetic data, is readily available and quite inexpensive. Even though several studies demonstrated promising positive aspects of Ivermectin treatment (115), being anti-inflammatory properties (116), prophylaxis treatment (NCT04668469), inhibition of virus replication (117), and patient survival (118), Ivermectin is not recommended as a COVID-19 drug by the FDA, EMA, and WHO. This is mainly explained by methodological limitations of various studies such as small sample sizes, the use of concomitant medications and controversial data (119). Further studies showed that up to 100-fold higher concentrations will be needed to achieve a concentration necessary for an antiviral effect (120). To finally clarify whether Ivermectin has beneficial effects in COVID-19 treatment the University of Oxford announced to add Ivermectin to the PRINCIPLE trial.

### Emerging Drug Candidates

Besides large drug screens, many research groups focused on the identification of novel virus-host interactions using genome wide-CRISPR/Cas screens (121–124). This revealed several factors that play a central role in various host pathways during infection, such as the cholesterol and phosphatidylinositol pathway, inflammatory signaling, cell cycle regulation or PML nuclear body formation and might represent new targets for antiviral approaches. One of the most prominent targets identified was the lysosomal transmembrane protein TMEM106B. Upon knock out (KO), SARS-CoV-2 replication was impaired (121). Consistent with this finding another research group further found TMEM106B expression to be induced during SARS-CoV-2 infection in patients and suggested a role of TMEM106B in virus entry (125). Another top-ranked protein was the membrane trafficking factor Rab7A. Knockout of Rab7A resulted in the sequestration of the SARS-CoV-2 receptor molecule ACE2 inside the cell and thereby led to reduced viral entry (121). In the light of emerging mutations that potentially reduce vaccine efficacy and the lack of specific antiviral therapeutics against SARS-CoV-2, these studies lay the ground for broadly active antivirals in the future.

## Interferons

IFNs are host cell-secreted signaling molecules that stimulate the concerted expression of antiviral restriction factors in infected and neighboring cells in an autocrine or paracrine manner, to restrict replication and prevent transmission of the invading pathogens. As other viruses, SARS-CoV-2 also antagonizes IFN-induction in infected cells, thereby calling for exogenous IFN-administration as a therapeutic strategy, which is already being exploited for treating diverse virus infections (126, 127). IFNs exist in three different types: Type-I, Type-II and Type-III, with distinct biological and antiviral properties. Despite its potential to antagonize IFN-induction, SARS-CoV-2 intriguingly demonstrates remarkable sensitivity towards lower doses of exogenous IFNs (128, 129), suggesting that early therapeutic application, and possibly prophylaxis with IFNs could be clinically promising. Thus, already early in the pandemic, the therapeutic potential of exogenous IFNs, mostly in combination with other treatments, was subject to clinical investigations.

### Type-I IFNs

Although many clinical studies have investigated the benefits of type I IFNs for the treatment of SARS-CoV-2 infections and COVID-19, the outcomes were highly diverse. This is facilitated by the differences in the study design, involving combinations with diverse other treatments as well as the selection of individual therapeutic entry points during the disease course, which strongly affect the antiviral actions of IFNs.

Two open-labelled trials assessed the efficacy of cotherapy of IFN-β1b (NCT04276688) or IFN-β1a (IRCT20151227025726N12) with LPV/r on mild-moderate COVID-19 hospitalized patients. The multicentre trial reported that the early triple cotherapy involving systemic administration of IFN-β1b with LPV/r was superior and safe compared to LPV/r alone, in attenuating the disease symptoms, and in reducing the clinical duration of viral shedding and hospital admission (73). Similarly, another single-centre retrospective trial (IRCT20151227025726N12) also reported favourable clinical outcomes of systemic IFN-β1a-addition with standard medications (LPV/r) and stated that although the mortality rates of the two groups were comparable, multivariate analysis showed that not-receiving IFN-β1a was significantly associated with all-cause mortality, alongside others factors like comorbidities and non-invasive ventilation (130), further corroborating the clinical benefits of early systemic IFN-β1b administration in cotreatment settings.

Only two associated clinical trials were performed on severe COVID-19 patients to assess the clinical benefits of IFN-β as therapeutic additive to standard medications (HCQ plus LPV/r or atazanavir/ritonavir) and reported favorable clinical outcomes. The trial administering IFN-β1b (IRCT20100228003449N27) reported that the test group had a shorter time to clinical improvement and discharge day and a lower 28-days mortality rate than the control group not receiving IFN-β1b (131). Although the other trial (IRCT20100228003449N28) reported that addition of IFN-β1a did not significantly accelerate the time to clinical improvement, it significantly accelerated the hospital discharge rate to day-14, lowered the 28-days mortality rate, and also improved the survival rates when administered during the early phase of the disease (132). In contrast to systemic administration, two clinical trials evaluated the therapeutic benefits of administering nebulized IFN early during mild COVID-19 and reported clinical benefits. An uncontrolled exploratory study found that nebulized IFN-α2b, alone or combined with Arbidol, reduced the time of quantifiable virus in the upper respiratory tract and reduced the duration of elevated inflammatory markers like IL-6 and CRP in the blood (133). In line with this, the multi-center trial (NCT04385095) on moderate hospitalized and ambulatory patients reported that nebulized IFN-β1a favored faster recovery than the control group, quantified based on the WHO Ordinal Scale for Clinical Improvement (OSCI) (134).

The timing of IFN-administration is likely a strong influential factor on the disease trajectory of COVID-19. Therefore, a retrospective multicenter cohort study carried-out in COVID-19 patients investigated the association between the timing of IFN-α2b administration with its clinical outcomes through regression analysis (135). Importantly, the study found that early administration was significantly associated with reduced in-hospital mortality and confers positive clinical outcomes. Similarly, a single-center retrospective-cohort clinical trial evaluated the therapeutic potency of IFN-β1b administration on moderate/severe pneumonia-positive hospitalized COVID-19 patients. The study reported that IFN-β1b-treatment did not significantly decrease in-hospital mortality at this disease stage, highlighting that IFN administration in the later stages of disease progression would have minimal to no significant clinical benefits (136). While several smaller clinical trials have reported mixed results based on diverse primary and secondary outcomes on the therapeutic benefits of IFN administration in single or cotreatment regimens in mild-moderate as well as on severe hospitalized and ambulatory patients, the WHO SOLIDARTY Consortium Trial is the largest clinical trial till-date that evaluated the therapeutic effects of IFN-β1a administration (and other drugs including HCQ, Remdesivir and LPV) in several thousand COVID-19 patients. The interim results from their multicenter trial at 405 hospitals in 30 countries conclude that there are no beneficial effects of IFN-administration, in terms of reducing in-patient mortality, initiation of mechanical ventilation, or hospitalization duration (Interim Results of WHO Solidarity Consortium Trial, Feb 2021).

Although more Type I IFNs exist in humans, currently only IFN-β (IFN-β1a and IFN-β1b) and IFN-α2 (IFN-α2b) as well as some engineered forms of IFNs are of clinical relevance. However, recent studies investigated the activity against SARS-CoV-2 of all twelve existing IFN-α subtypes, and demonstrated highly efficient and subtype-specific antiviral properties (137, 138), which strongly emphasizes that IFN-α subtypes should be included in clinical investigations in order to increase our IFN-therapeutic arsenal.

### Type-III IFNs

Encouraged by the preclinical evidences on the inhibitory potential of IFN-λ1 on SARS-CoV-2 replication *in vitro* and *in vivo* (139, 140), two Phase-II clinical trials evaluated the therapeutic efficacy of single-dose subcutaneous administration of pegylated (peg) IFN-λ1a in ambulatory uncomplicated COVID-19 patients. While the COVID-LAMBDA trial (NCT04331899) reported that the treatment regimen neither reduced the duration of virus shedding, nor alleviated disease symptoms (141), the ILIAD trial (NCT04354259) reported that the treatment regimen significantly accelerated virus clearance as quantified by qRT-PCR of SARS-CoV-2 vRNA in nasopharyngeal swabs, concluding that peg-IFN-λ1a administration has therapeutic benefits to shorten virus shedding and prevent clinical progression (142). Currently ongoing trials aim to further evaluate the therapeutic efficacy of single dose (NCT04343976) or double-dose (NCT04534673 and Phase-IIb of NCT04354259) subcutaneous administration of peg-IFN-λ1a in hospitalized moderate COVID-19 patients, in terms of clinical time to reach negative qRT-PCR outcome, mortality rate and recovery period. Results from these trials would likely throw light for choosing optimal treatment regimen to achieve favorable clinical outcomes in hospitalized patients.

### Other IFNs

Several clinical trials investigated the therapeutic benefits of other non-conventional IFN subtypes. For instance, a non-randomized clinical trial (ChiCTR2000030262) on mild COVID-19 patients tested the therapeutic efficacy of aerosol inhalation of IFN-κ with Topical Trefoil Factor Family-2 (TFF2), which is a peptide isoform demonstrating proven therapeutic benefits against gastrointestinal disorders associated with mucosal damage (143). This treatment regimen significantly improved clinical outcomes, including cough-relief, CT-imaging, and vRNA negativity, altogether favoring an early discharge from hospitalization (144). Another Phase-II clinical trial (ChiCTR2000029638) was conducted on moderate to severe COVID-19 patients to evaluate the safety and efficacy of Recombinant Super-Compound Interferon (rSIFN-co) versus traditional nebulized IFNα-2b, alongside standard antivirals (LPV/r or Umifenovir) (145). The trial observed that co-treatment with rSIFN-co was safer and more efficacious than traditional IFNα-2b for treating moderate to severe COVID-19, warranting further clinical trials of rSIFN, alone or in combination with other antiviral agents.

While the concept of cotreatment classically revolves around the use of two different drugs, or currently with a drug combined with an IFN-type, to accomplish improved clinical benefits at lower doses of the individual agents, encouraging results came out of clinical trials evaluating combinations of two different IFN types. For instance, based on the synergic inhibition of SARS-CoV-2 replication by type-I and type-II IFN combinations (146–149), a controlled clinical trial was conducted to assess the efficacy and safety of subcutaneous IFN-α2b and IFN-γ coadministration along standard medication (LPV/r), in SARS-CoV-2-positive hospitalized patients (150). The trial reported that IFN-α2b+IFN-γ cotreatment eliminated the virus earlier than IFN-α2b standalone treatment, although the latter also demonstrated appreciable efficacy for SARS-CoV-2 treatment.

### IFN Prophylaxis

While several trials evaluated the therapeutic benefits of IFN administration, preliminary trials are also being deployed on a prophylactic setting to prevent infections. Inspired by the preclinical studies on the high maintenance of IFN concentration as nasal droplets (129, 151), a prospective open-label clinical trial (NCT04320238) evaluated the prophylactic administration of recombinant human IFN-α (rhIFNα) nasal droplets on medical staff in Hubei, China. From the trial the authors concluded that prophylactic application was remarkably effective in preventing COVID-19 in medical staff that are at a high risk of acquiring infection, which was confirmed by negative pulmonary CT scans and the absence of other clinical symptoms (152). Along the same line, the PROTECT Trial (NCT04344600) currently evaluates the prophylactic potential of a single subcutaneous peg-IFN-λ1 administration on non-hospitalized individuals that are at a high risk of SARS-CoV-2 infection due to household exposure.

## Immunomodulators

Because the development of COVID-19 is strongly driven by a dysregulated, often overshooting immune response, different immune modulatory treatments are considered for early and late phases of COVID-19 and several promising approaches for immuno-therapeutic treatments are under clinical investigations.

### Dexamethasone

Glucocorticoids such as Dexamethasone constitute a frequently used class of anti-inflammatory drugs and have been used to treat chronic rhinosinusitis, chronic respiratory diseases or autoimmune disorders like rheumatoid arthritis (153, 154). Apart from their extensive use, they are very well-studied, widely available and also inexpensive (155). Dexamethasone disrupts inflammatory processes by decreasing the peripheral concentration and function of immune cells and limits the release of cytokines such as TNF-α and IL-1 from macrophages and other antigen-presenting cells (156, 157). The preliminary results from RECOVERY trial indicated that an oral or intravenous Dexamethasone dose of 6 mg given once daily for up to 10 days resulted in 35% less COVID-19 related deaths in patients on ICU who require mechanical ventilation and 20% less in non-ventilated patients on oxygen therapy (158). In addition, hospitalization time was significantly reduced. In contrast, there was no benefit for patients who do not receive respiratory aid. Based on this preliminary report the FDA and EMA endorsed the use of Dexamethasone for treatment of COVID-19 in late 2020. Later trials in COVID-19 patients with ARDS support this finding and report reduced overall mortality, and an increase in ventilation-free days (159, 160) Since November 2020, the Adaptive COVID-19 Treatment Trial 4 (ACTT-4) is active for a Phase-III study evaluating combinational therapy using Dexamethasone and Remdesivir in hospitalized patients (NCT04640168) as previous studies showed that combining Dexamethasone and Remdesivir enhances the positive effect of Remdesivir, leading to reduced mortality in hospitalized patients (161). Despite the promising results in ARDS and COVID-19 patients, there is still uncertainty about the overall effectiveness of glucocorticoids for COVID-19 patients as corticosteroids can also induce unwanted adverse effects, such as hyperglycemia, bacterial, fungal and viral infections, skin changes, adrenal suppression, myopathy and effects on wound healing and bone metabolism. Furthermore, there are potential ophthalmic, gastrointestinal, cardiovascular and psychiatric side effects (154).

### IL-6 Receptor Antagonists

The proinflammatory cytokine IL-6 is one of the hallmark cytokines upregulated during severe COVID-19 and represents a predictive marker for the need of mechanical ventilation in accordance with CRP levels (162). Based on the critical role of IL-6 signaling, therapeutic approaches employed strategies to inhibit IL-6 signaling to reduce inflammation.

Tocilizumab and Sarilumab are both monoclonal antibodies, which inhibit the membrane-bound and the soluble form of the IL-6 receptor (163). Tocilizumab is already widely used for the treatment of diverse chronic and autoinflammatory diseases like rheumatoid arthritis, systemic juvenile idiopathic arthritis and others (164–167). The benefit of Tocilizumab treatment for hospitalized COVID-19 patients has been investigated in several clinical studies, however, early clinical trials conducted in Brazil were terminated due to safety issues (168). The first successful study was conducted in China with a cohort of only 15 COVID-19 patients. After administration of Tocilizumab, IL-6 levels first spiked but then decreased in 66.7% of the patients. A repeated dose of Tocilizumab was suggested to be more likely to improve the status of critically ill patients (169). Another study showed that Tocilizumab immediately improved the clinical outcome in severe and critical COVID-19 patients while no adverse side effects were registered (170). Since 2020, Roche is conducting three trials investigating the effect of Tocilizumab on COVID-19. The COVACTA trial showed a reduction in hospitalization time in COVID-19 patients treated with Tocilizumab, but there was no significant clinical improvement nor mortality reduction [NCT04320615; (171)]. The EMPACTA trial dealt with ethnic minorities and observed a reduction of mechanical ventilation by day 28, but also no difference in mortality between treated and non-treated patients [NCT04372186; (172)]. Lastly, the REMDACTA trial was enrolled to evaluate the safety and efficacy of the combination of Tocilizumab and Remdesivir in patients hospitalized with severe COVID-19 pneumonia, but did not meet the primary endpoint of earlier hospital discharge with treatment (NCT04409262). Additionally, other studies (NCT4356937, NCT04331808, NCT04346355) could also not show a benefit of Tocilizumab in preventing death (173–175). Critics also point out the high price of Tocilizumab, turning it to a drug only available in wealthy countries instead of a global treatment approach (176). Despite these disappointing results, the large-scale REMAP-CAP and RECOVERY trials concluded that for hospitalized, critically-ill COVID-19 patients, treatment with either Tocilizumab or Sarilumab improved the survival and other clinical outcomes, suggesting a superiority to standard care and an additive benefit when administered in combination with Dexamethasone (REMAP-CAP NCT02735707; RECOVERY Collaborative Group NCT04381936). Since 26^th^ of June 2021, the FDA gave an EUA for Tocilizumab (Actemra) for the treatment of hospitalized pediatric or adult COVID-19 patients, when they are in need for supplemental oxygen, mechanical ventilation or ECMO. Later, at the 6th of July 2021, the WHO officially recommended the usage of IL-6 receptor blockers based on a network meta-analysis with data from clinical trial investigators in 28 countries (WHO).

### Anti-IL-6 Antibodies

Siltuximab is a human-mouse chimeric monoclonal antibody against IL-6, which is approved for treatment in HIV-negative patients with multicentric Castleman’s disease (177). Preliminary data from a study with 21 COVID-19 patients suffering from ARDS indicated an improvement of the clinical condition after treatment with Siltuximab in 7 of the patients, while 9 patients stabilized with no clinically relevant improvement and the condition of the remaining 5 patients worsened [NCT04322188; (178)] Ongoing studies investigate the efficacy and safety of Siltuximab for COVID-19 treatment in comparison and in combination with corticosteroids such as methylprednisolone (NCT04329650, NCT04486521).

### MAPK p38 Inhibitors

The mitogen-activated protein kinase (MAPK) p38 is associated with dysbalanced inflammatory processes in different pathologies and well-studied in animal models of acute lung and myocardial injury (179, 180). Mechanistically, p38-mediated phosphorylation of downstream kinases regulates the expression of proinflammatory cytokines such as IL-6, TNF-α and IL-1β. In the context of a dysbalanced immune response, such as in severe COVID-19 (11, 181), overexpression of these cytokines is suspected to contribute to organ damage and the described immunopathologies, suggesting that therapeutic interventions that reduce the expression of these cytokines could provide beneficial treatments for COVID-19. Recent work has demonstrated that p38 is activated during SARS-CoV-2 infection *in vitro* (180, 182, 183) and that blocking of p38 signaling by using pharmacological inhibitors that bind to the ATP binding pocket of this kinase resulted in decreased cytokine levels *in vitro* (182). Based on this observation Grimes et al. suggested p38 signaling as a promising therapeutic target for immunomodulatory therapeutic approaches of COVID-19 (184). In August 2020, Fulcrum Therapeutics initiated a Phase-III clinical trial with losmapimod, a clinically pre-evaluated but non-licensed, orally available p38 inhibitor, to investigate its effect on mortality and clinical outcome in hospitalized patients with moderate COVID-19, which are at risk to develop severe disease (NCT04511819). The results of this study are not yet released.

### JAK Inhibitors

The Janus Kinase (JAK) family members play an essential role for the intracellular cytokine receptor signaling (185). They closely interact with signal transducer and activator of transcription (STAT) proteins and thereby contribute to inflammation and antiviral responses (186). Already in February 2020, Richardson et al. introduced Baricitinib, a JAK kinase inhibitor, to be trialed in COVID-19 patients (187) as it showed inhibitory properties against IL-6 and STAT3 phosphorylation and is approved for treatment of rheumatoid arthritis (188). Consistently, rhesus macaques had reduced inflammation in the lung upon treatment during SARS-CoV-2 infection, however, virus titers remained unaffected, demonstrating that it has immunomodulatory but not antiviral properties (189). However, in combination with Remdesivir, Baricitinib reduced the recovery time of patients receiving high-flow oxygen or noninvasive ventilation (190). Of note, patients also underwent standard treatment with corticosteroids, which limits the validity of the effect of Baricitinib. Nevertheless, on November 19th 2020, Baricitinib received EUA from the FDA in combination with Remdesivir (191).

Similar to Baricitinib, the JAK inhibitor Tofacitinib is also approved as an anti-rheumatic drug (192). When applied to COVID-19 patients with high levels of CRP within the TOFA-COV-2 trial, Tofacitinib treatment lowered the lung damage and CRP levels, increased the oxygen saturation and thus was suggested as an effective medication for managing the cytokine levels during COVID-19 (193) (NCT04750317).

In June 2021, a clinical trial using JAK inhibitor Ruxolitinib for treatment of COVID-19 patients was completed (RUXCOVID NCT04362137). Ruxolitinib is already an approved medication for myelofibrosis since 2012 (194). Unfortunately, the RUXCOVID trial sponsored by Novartis Pharmaceuticals could not meet the primary outcome of reducing the number of COVID-19 patients with severe complication such as ICU care, mechanical ventilation or death (NCT04362137). Before, a small study in 2020 indicated significant clinical improvement of patients with hyperinflammation during COVID-19 without signals of Ruxolitinib-induced toxicity (195). Based on these results, a Phase-II clinical trial has been completed, but only comprised 3 patients and did not publish any results (NCT04331665). More studies are currently recruiting participants to further assess the efficiency of Ruxolitinib for treating COVID-19 patients suffering from hyperinflammation and dysregulated immune responses (NCT04334044, NCT04338958, NCT04581954).

### Emerging Immunomodulatory Treatment Strategies

Based on the rationale that the reduction of the overly activated proinflammatory response during COVID-19 improves clinical outcomes, many studies investigate approaches to target proinflammatory processes and other cytokines. Similar to the mechanism of IL-6 antagonists, antagonists of the IL-1 family and TNF blockers were investigated. The IL-1β antagonist Anakinra could successfully ameliorate the cytokine storm in infected patients with severe sepsis (196). A completed meta-analysis of clinical publications could already demonstrate a reduced mortality rate in treated patients (197),NCT04443881). Hence, new clinical trials with Anakinra are planned, injecting 100 mg Anakinra subcutaneously for up to 10 days (NCT04357366, NCT04643678, NCT04362111 (198, 199). Other potential measures focus on targeting mononuclear macrophage recruitment and their function or even stem cell therapy (200, 201) NCT04366063; NCT04486001; NCT04252118; NCT04437823), but there is no clinical information yet whether this is of benefit in COVID-19 patients.

## Long COVID

In addition to the treatment of acute COVID-19, the need for efficient therapeutic options for the long-term consequences of the infection rapidly increases as many infected individuals suffer from Long COVID (202). Reports on the symptoms of Long COVID range from pulmonary manifestations such as shortness of breath, persistent cough and declined lung function to extrapulmonary manifestation like fatigue, anxiety and depression, memory loss, anosmia as well as declined kidney function, gastrointestinal complaints and heart damage (203–206).

Whilst this condition was first described as post COVID-19 syndrome or chronic COVID-19, the patient-derived name Long COVID or Long-haul COVID has recently been favored within the literature to draw attention to its severity (207, 208). The National Institute for Health and Care Excellence (NICE) further divides Long COVID into three separate groups according to the time of symptom duration: (I) acute COVID-19 (for up to 4 weeks) (II) ongoing symptomatic COVID-19 (4 to 12 weeks) (III) post-COVID-19 syndrome (more than 12 weeks) (COVID-19 Rapid Guideline, Dec 2020) (209, 210).

The knowledge of Long COVID is still rather small and it is not even clear if all reports of long-lasting effects on patient´s health describe the same entity (211). There are multiple different hypotheses of what may trigger Long COVID that still remain to be investigated. Ideas range from a post-viral syndrome similar to myalgic encephalomyelitis/chronic fatigue syndrome (ME/CFS), a potential persistence of the virus in certain organs, an auto-immune triggered disease mechanism and post-intensive care syndrome (212). It was recently shown that a median of 4 months after COVID-19 diagnosis the SARS-CoV-2 N-protein could be detected within enterocytes in 5 out of 14 intestinal biopsies illustrating that antigen-persistence could potentially play a role in the development of Long COVID (213). Another disease mechanism that was recently suggested is that of an orthostatic intolerance syndrome caused by a disruption of the autonomic nervous system through infection with the SARS-CoV-2 virus (214). In 31 patients suffering from Long COVID symptoms two to seven different functional autoantibodies against G-coupled receptors could be detected illustrating that autoimmunity might play a role in the development of Long COVID (215). There is also a lack of information on how to best treat Long COVID patients. In a recent not yet peer-reviewed prospective observational study receiving the vaccination seemed to be beneficial to the recovery process at a median of 32 days after the vaccination. In 5.6% of the 44 vaccinated participants, symptoms increased, whilst 14.2% of the 22 not-vaccinated participants reported such an increase. Symptom resolution was experienced in 23.2% of those vaccinated in comparison to 15.4% of those not vaccinated. A difference between the Biontech-Pfizer or the AstraZeneca vaccine used in this study could not be observed (216).

The largest survey about the impact of different vaccines on the symptoms of patients suffering from Long COVID was conducted by the patient advocacy group LongCOVIDSOS *via* an online questionnaire with 900 participants. In 56.7% of all participants, symptoms overall improved after vaccination, while 18.7% experienced a deterioration. Only in 2.9% of participants all symptoms worsened. Interestingly, the Moderna mRNA vaccine appeared to be especially beneficial in relieving symptoms (217). Accordingly, it appears that receiving the vaccine is at least not harmful for those suffering from Long COVID. It has recently been suggested that an infection with SARS-CoV-2 results in an increasing amount of double negative B memory cells, which are dysfunctional. RNA vaccination reduced the number of those cells. This could be a potential reason why RNA vaccination seems to relieve the symptom burden in some patients (218).

To gain more insight into Long COVID the post-hospitalization study (PHOSP-COVID) has been set up in the UK and will recruit 10.000 patients that have been hospitalized with COVID-19, in order to better understand the short-, medium- and long-term consequences of infection *via* collecting patient data and material for one year after discharge from hospital (www.phosp.org).

## Concluding Remarks

**Figure 1:**
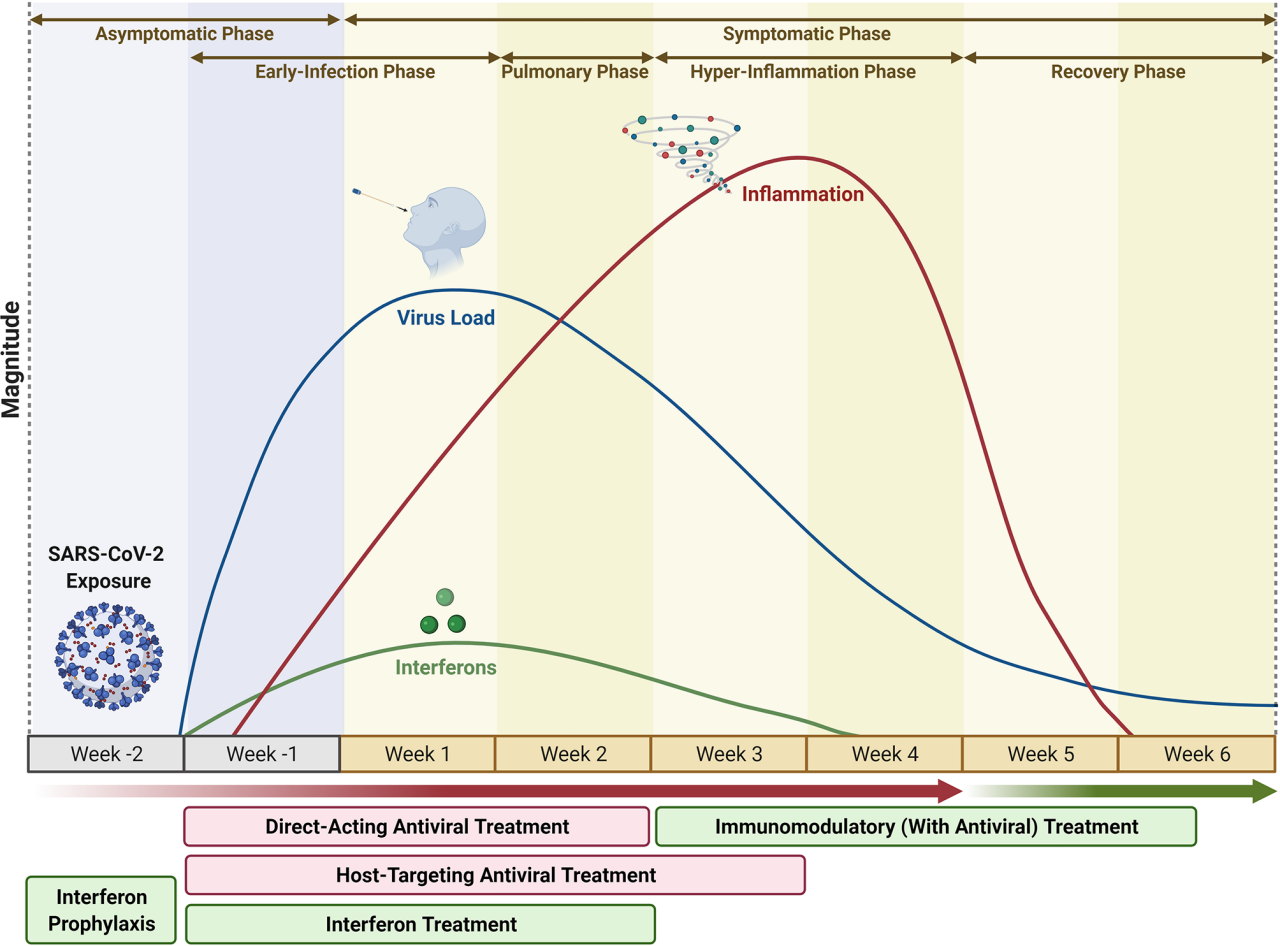
COVID-19 Progression vs Treatment Options. The infection phase of COVID-19 begins 1-week post exposure to SARS-CoV2 (Asymptomatic phase) and lasts for 4 weeks from the time of onset of symptoms, marked by a steady increase and resolution in virus titer between the first 2 weeks, during which Direct-Acting Antivirals (DAA), alone or in combination with Host-Targeting Antivirals (HTA) or Recombinant Interferons, could be potential treatment options. Week-2 until week-4 marks the resolution of infection phase, and onset of pulmonary phase (week-1, week-2) followed by inflammatory phase (week-3, week-4), during which Immunomodulators, mostly in combination with Antivirals, could be potential treatment options. Interferon Prophylaxis could be a preventive strategy on suspected exposure to SARS-CoV2 during the asymptomatic, pre-infection phase. (Timecourse not to be exactly scaled; varies depending on patient heterogeneity and virus variant). Figure modified from (135), and created with BioRender.com.

More than one and a half years into the SARS-CoV-2 pandemic, the treatment options for severe COVID-19 are still very limited. However, as this review highlights, the number of new drug candidates that are in clinical development or have already received EUA and are on the way to full approval is constantly growing. This include the immunomodulatory JAK inhibitor Baricitinib and the IL-6 receptor blocker tocilizumab, which both targets prevent signaling pathways that are involved in the expression levels of proinflammatory cytokines. The success of such approaches in the treatment of COVID-19 emphasizes that immunomodulatory and not primarily antiviral treatments are the key to COVID-19 therapies as the late-stage clinical manifestation of this disease is dominated by imbalanced inflammatory responses rather than by virus replication itself (**Figure 1**).

However, antiviral approaches aiming to reduce viral replication are similarly required to prevent the development of COVID-19 which is most successful in the earlier stages of the disease and can prevent further systemic dissemination of the virus and development of systemic inflammation. Under these considerations, combination of antiviral and immunomodulatory drugs or drugs that comprise both actions offers the broadest treatment window and appears most promising to achieve positive clinical outcomes in COVID-19 patients.

Clearly, the majority of the new therapeutic options are constituted of repurposed drugs or derived from classical therapeutic strategies such as treatment with convalescent sera or monoclonal antibodies, that are already in clinical use for other diseases and for which the route of administration, safety and side effects in humans is already well characterized. Nevertheless, as this review summarizes, there are also novel strategies, such as the ACE2-Fc fusion protein. Next to clinical therapeutics, home-treatment options are also being assessed for several drugs with the aim to reduce the time of viral shedding and the burden of milder disease. Another advantage of an increased arsenal of treatment options against SARS-CoV-2 and COVID-19 can be seen in potential to prevent the development of long-term complications like Long COVID.

## Author Contributions

LB is responsible for the concept and structure of the review. SK, DC, JJ, AF, BC, and JL collected the literature articles and drafted the individual sections. SK edited the initial draft and designed the final figure. LB and SL reviewed and revised the final draft. All authors contributed to the article and approved the submitted version.

## Funding

This work was financially supported by the *German Research Foundation (DFG)* through the *Clinical Research Unit CRU342* (for Project-P6 to LB and SL); the *Federal Ministry of Education and Research (BMBF)* for the project CoIMMUNE (Grant No. 01KI20218 to LB), and through the *Network University Medicine (NUM)* for the COVID-19 project Organo-Strat (Grant No. 01KX2021 to LB and SL); and by the Faculty of Medicine, University of Muenster, through the *Interdisciplinary Centre for Clinical Research (IZKF)* (Grant No. Bru2/015/19 to LB) and the *Innovative Medizinische Forschung (IMF)* (Grant No. BR111905 to LB). DC is financially supported through a Study Abroad Program by the *Ministry of National Education* (Law: 1416), *Republic of Turkey*. 

## Conflict of Interest

SL is a founder, shareholder and board member of Atriva Therapeutics GmbH, Tübingen, Germany, a pharmaceutical company developing novel HTA against respiratory viral diseases including COVID-19.

The remaining authors declare that the research was conducted in the absence of any commercial or financial relationships that could be construed as a potential conflict of interest.

## Publisher’s Note

All claims expressed in this article are solely those of the authors and do not necessarily represent those of their affiliated organizations, or those of the publisher, the editors and the reviewers. Any product that may be evaluated in this article, or claim that may be made by its manufacturer, is not guaranteed or endorsed by the publisher.

## Abbreviations

ACTT, Adaptive COVID-19 Treatment Trial; ADE, Antibody-dependent Infection Enhancement; ARDS, Acute Respiratory Distress Syndrome; CFS, Chronic Fatigue Syndrome; COPD, Chronic Obstructive Pulmonary Diseases; DAA, Direct-Acting Antivirals; eEF1A, eukaryotic translation Elongation Factor 1 Alpha; EMA, European Medicines Agency; EUA, Emergency Use Authorization; FDA, Food and Drug Administration; FIASMA, Functional Inhibitors of Acid Sphingomyelinase; GBPA, 4-(4-Guanidinobenzyloxy)Phenylacetic Acid; HCQ, Hydroxy-Chloroquine; hrsACE2, Recombinant human soluble ACE2; LPV/r, Lopinavir/ritonavir; ME, Myalgic Encephalomyelitis; NICE, National Institute for Health and Care Excellence; NSP, Non-Structural Proteins; ORF, Open Reading Frames; OSCI, Ordinal Scale for Clinical Improvement; RdRp, RNA-dependent RNA Polymerase; rhIFNα, Recombinant Human IFN-α; rSIFN, Recombinant Super-Compound Interferon; HTA, Host-Targeting Antivirals; SARS-CoV-2, Severe Acute Respiratory Syndrome Coronavirus 2.
